# Oxygen Saturation Targeting in the Neonatal Intensive Care Unit

**DOI:** 10.3390/jcm14113975

**Published:** 2025-06-04

**Authors:** Faeq Almudares, Bheru Gandhi, Jonathan Davies, Xanthi Couroucli, Natalie Villafranco, Nidhy Paulose Varghese, Milenka Cuevas Guaman, Charleta Guillory, Binoy Shivanna

**Affiliations:** 1 Division of Neonatology, Department of Pediatrics, Baylor College of Medicine, Houston, TX 77030, USA; faeq.al-mudares@bcm.edu (F.A.); bbgandhi@texaschildrens.org (B.G.); jldavies@texaschildrens.org (J.D.); xicourou@texaschildrens.org (X.C.); mxcuevas@texaschildrens.org (M.C.G.); cxguillo@texaschildrens.org (C.G.); 2 Division of Pulmonology, Department of Pediatrics, Baylor College of Medicine, Houston, TX 77030, USA; nxvillaf@texaschildrens.org (N.V.); npvarghe@texaschildrens.org (N.P.V.)

**Keywords:** neonatal SpO_2_, bronchopulmonary dysplasia, pulmonary hypertension, retinopathy of prematurity

## Abstract

Oxygen (O_2_) is vital for cellular development, function, proliferation, and repair, underscoring its critical role in organogenesis. Both hypoxia (reduced tissue O_2_) and hyperoxia (excess tissue O_2_), when prolonged, can trigger inflammation and oxidative stress, contributing to acute and long-term cardiopulmonary and neurodevelopmental morbidity. In sick neonates, immature defense mechanisms and coexisting morbidities demand nuanced and sometimes opposing strategies for O_2_ saturation targets and therapeutic titration. Most current neonatal O_2_ targeting guidelines are based on animal models or small clinical studies, resulting in recommendations with limited evidence. This narrative review aims to provide an updated overview of the physiological roles of O_2_ in development, its delivery and consumption, approaches to O_2_ saturation monitoring, and therapeutic targeting in neonates under both normal and pathological conditions. We also highlight key knowledge gaps and propose directions for future research on neonatal O_2_ saturation targeting.

## 1. Introduction

Identified in the 1970s by Joseph Priestley [1], oxygen (O_2_) is essential for the development, function, proliferation, and repair of cells and tissues, emphasizing the importance of its availability in the development of organs. O_2_ supports energy requirements through cellular respiration. Therefore, it is also considered a macronutrient, as it nourishes the organism for survival [2].

O_2_ therapy has been widely utilized in neonatal intensive care units to treat hypoxic respiratory failure; however, there is a scarcity of randomized controlled trials assessing its optimal use for maximizing benefits and minimizing risks. Both hypoxia (lower O_2_ at the tissue level) and hyperoxia (increased O_2_ at the tissue level) for prolonged periods induce inflammation and oxidative stress and cause short- and long-term cardiopulmonary and neurodevelopmental morbidities [3,4,5,6]. Additional co-morbidities and immature defense mechanisms in sick infants [3] necessitate diverse and sometimes contradictory approaches to target O_2_ saturation. For instance, higher O_2_ saturation targets might be needed for conditions like pulmonary hypertension (PH), whereas it is advisable to avoid higher O_2_ saturation in infants with severe retinopathy of prematurity (ROP). These factors create a unique and demanding set of challenges for medical providers caring for premature infants. Most guidelines adopted by various international organizations for the neonatal population are based on animal studies or small clinical trials, leading to recommendations founded on low levels of evidence and resulting in varying grades of recommendation strength [7,8,9,10,11,12].

Several reviews have been published on O_2_ saturation targets in neonates. However, most of these studies have been narrow in scope or focused on specific neonatal diseases. Hence, the major objective of this narrative review is to provide an updated comprehensive overview of the essential functions of O_2_ during development, O_2_ delivery and consumption, O_2_ saturation assessment, and O_2_ saturation targeting in the physiological and pathological states of neonates. We also highlight key knowledge gaps and outline future directions to guide research on optimizing O_2_ saturation targeting for this vulnerable population.

## 2. Literature Search Methods

We searched the PubMed database through the time period 1960 to the present. The following mesh terms without any language restrictions were included in our search: “Oxygen” AND “Development” OR “Oxygen transport OR delivery OR consumption” AND “Neonates OR Infants” OR “Oxygen Saturation” AND “Fetus” OR “Oxygen Saturation” AND “Extrauterine Transition” OR “Oxygen Saturation Targets” AND “Neonates OR Infants” OR “Oxygen Saturation OR Oxygen Partial Pressure” AND “Assessment” AND “Neonates OR Infants” OR “Oxygen Saturation” AND “Preterm Infants” OR “Oxygen Saturation” AND “Bronchopulmonary Dysplasia” OR “Oxygen Saturation” AND “Pulmonary Hypertension” AND “Neonates OR Infants” OR “Oxygen Saturation” AND “Bronchopulmonary dysplasia” AND “Pulmonary Hypertension” OR “Oxygen Saturation” AND “Retinopathy of Prematurity” OR “Oxygen Saturation Targets” AND “Artificial Intelligence” AND “Neonates OR Infants”. The exclusion criteria included case reports, case series, non-English articles, and studies that did not contribute relevant information to this review. Two authors (FA and BS) independently screened the titles and abstracts of the studies to determine their eligibility. We obtained the full-text version for further evaluation and resolved any discrepancies through discussion.

## 3. O_2_ and Development

Embryogenesis is a dynamic process that results in the development of organs. O_2_ is essential for the development, function, proliferation, and repair of cells and tissues, emphasizing the importance of its availability for organ development. The presence of O_2_ in the Earth’s atmosphere has supported these cell- and tissue-protective effects and facilitated the evolution of multicellular life, including humans [13]. During development, O_2_ levels can vary based on the needs of the developing embryo [2]. The O_2_ tension in the mammalian oviduct, where embryogenesis begins, is approximately 5–7% versus 21% found in the normal atmospheric environment [14]. Low O_2_ tension during this early embryogenesis phase is crucial for maintaining the balance between reactive oxygen species (ROS) production and antioxidant enzyme activity, creating an ideal environment for cell development [15].

Hypoxia-inducible factors (HIF) and their downstream target genes, which promote cell proliferation and preserve cells in an undifferentiated state, primarily regulate the low O_2_ tension phase of chondrogenesis [16]. Following placental formation, O_2_ levels increase to optimize the O_2_ demand of the growing fetus [14]. Alterations in O_2_ concentrations during embryogenesis can cause oxidative stress and metabolic dysfunction, disrupt biological processes, and negatively affect organ development [17,18,19,20,21,22].

## 4. O_2_ Transport, Delivery, and Consumption

In humans, most O_2_ is consumed for cellular mitochondrial respiration, where aerobic metabolism of glucose generates adenosine-5′-triphosphate (ATP), the primary energy source for cellular physiological processes. Due to the constant metabolic need for O_2_ and the lack of significant storage in the body, a continuous supply of O_2_ is essential for survival [2]. Although O_2_ can be stored in skeletal muscles via myoglobin, this form of storage is minimal and is primarily observed in marine animals [23].

Due to its low water solubility, O_2_ is primarily transported to tissues by hemoglobin through a series of steps [24]: first, O_2_ is delivered from the atmosphere to the alveoli in the lungs; it then diffuses across the alveolar epithelium and capillary endothelium into the circulation, where it rapidly and reversibly binds to hemoglobin in the erythrocytes. The hemoglobin-O_2_ binding affinity is influenced by the factors listed in Table 1, which shift the O_2_ dissociation curve to the right or left, thereby increasing or decreasing the P50 value (Table 1) [25]. Each hemoglobin complex carries up to four O_2_ molecules [25]. Thus, the majority of O_2_ is transported via hemoglobin, and only a minor quantity of O_2_ is transported in the dissolved form. O_2_-bound hemoglobin is then transported by blood flow to various tissues, where O_2_ is finally delivered to the mitochondria via diffusion down the concentration gradient. The progressively lower partial pressures of O_2_ (PO_2_) in the capillaries, tissues, cells, and mitochondria compared to those in the atmosphere, alveoli, and larger blood vessels facilitate the final step of O_2_ transport [26,27]. Additionally, variations in vascular architecture, cell types and density, metabolic activity, and O_2_ demand across different tissues contribute to the heterogeneity in normal PO_2_ levels both between and within organs and tissues throughout the body [26,27,28]. Erythrocytes are ideal O_2_ carriers because they lack mitochondria and rarely consume O_2_ [28].

The rate at which O_2_ is transported from the lungs to the tissue microcirculation is known as O_2_ delivery (DO_2_), which is primarily determined by arterial O_2_ content (CaO_2_) and cardiac output [29,30]. O_2_ consumption (VO_2_) is the rate at which O_2_ is removed from the blood and used by the tissues, while O_2_ extraction represents the fraction of arterial O_2_ removed from the blood as it circulates through the tissue microcirculation. DO_2_ exceeds the basal requirements by approximately threefold under homeostatic conditions [30]. Tissue O_2_ needs may exceed supply when metabolic demands increase, and DO_2_ decreases due to various pathological conditions. Tissues initially adapt to decreased DO_2_ by increasing O_2_ extraction [25]. However, this adaptive response fails beyond a certain low DO_2_ level. The DO_2_ level at which increased O_2_ extraction cannot meet metabolic needs is known as the critical DO_2_ level. In cases of increased metabolic demand, the body’s primary compensatory strategy is to enhance DO₂ rather than relying solely on increased O_2_ extraction [29]. The fetus lives in a relatively hypoxic environment, which stimulates erythropoietin production and induces erythropoiesis to boost O_2_-carrying capacity. The high affinity of fetal hemoglobin for O_2_ is ideal for efficient O_2_ uptake from the placenta to the fetus. [31,32].

## 5. Assessment of O_2_ Saturation and Partial Pressure of O_2_

O_2_ saturation can be measured invasively in the arterial system (SaO_2_) via arterial blood sampling or noninvasively by pulse oximetry (SpO_2_). The SaO_2_ value is determined by a co-oximeter based on light absorption at several wavelengths, whereas the SpO_2_ value is determined by a pulse oximeter based on light absorption at two wavelengths corresponding to the peaks of oxygenated and deoxygenated hemoglobin [33]. The advantage of invasive sampling measurement is that it also estimates pH, PaO_2_, and PaCO_2_, which are necessary for monitoring critically ill patients with cardiorespiratory and metabolic problems. The accuracy of these values varies depending on the sampling site, with arterial samples providing the most accurate values. The advantages of noninvasive pulse oximetry monitoring include access to instant and continuous SpO_2_, cost-effectiveness, and the absence of pain and discomfort associated with invasive monitoring. Further, SpO_2_ values closely correlate with SaO_2_ (≤2%), providing an accurate estimation of arterial O_2_ saturation [25]. However, the accuracy of pulse oximetry-based SpO_2_ values may decline with poor peripheral perfusion, lower O_2_ saturation values, and the presence of methemoglobin and carboxyhemoglobin [25]. The partial pressure of O_2_ in the arteries (PaO2) is estimated mainly by invasive sampling of arterial blood. The partial pressure of O_2_ can also be noninvasively measured transcutaneously (transcutaneous partial pressure of O_2_ or PTcO_2_) by applying electrodes to the skin [34]. The electric current generated by the O_2_ at the electrode is transformed into pressure measurements. The accuracy of noninvasive pO_2_ readings can be affected by sensor temperature, peripheral perfusion, anemia, acidosis, and skin thickness [34].

Recently, near-infrared spectroscopy (NIRS) has been increasingly used in infants to noninvasively measure tissue oxygenation in several organs, including the lungs [35,36,37,38,39,40,41]. Based on oxyhemoglobin and reduced hemoglobin concentrations measured by near-infrared light in the tissues, NIRS calculates regional tissue O_2_ saturation. The NIRS value is a composite measure of the values obtained in the arterial, venous, and capillary beds of the tissue [42]. However, the application of the device, its recordings, and result interpretation need to be standardized and rigorously validated [42] before NIRS monitoring can be widely adopted as a standard of care for critically ill infants.

## 6. O_2_ Saturation and Transition to Extrauterine Life in the Delivery Room

At birth, the lungs undergo significant changes to adapt to the extrauterine environment. Placental separation leads to an elevation in systemic vascular resistance, accompanied by a reduction in pulmonary vascular resistance as ventilation begins. These changes, along with the establishment of breathing patterns and the clearance of fetal lung fluid, result in increased pulmonary blood flow and the establishment of ventilation and oxygenation [31,43]

Healthy neonates who do not need respiratory support or O_2_ supplementation gradually increase their O_2_ saturation after birth, reaching 90% within 5–8 min of life [44,45,46,47]. Infants born vaginally achieve 90% O_2_ saturation faster than those born via cesarean section. The median O_2_ saturation at 1 min of life is 63%. Spontaneously breathing term newborns who undergo delayed cord clamping attain higher O_2_ saturation than those with immediate cord clamping [48]. The American Academy of Pediatrics (AAP) and American Heart Association (AHA) recommend the following target O_2_ saturations for newborns during delivery room resuscitation: at 1 min: 60–65%; at 2 min: 65–70%; at 3 min: 70–75%; at 4 min: 75–80%; at 5 min: 80–85%; and at 10 min: 85–95%. If positive pressure ventilation is needed, an initial O_2_ concentration of 21% should be used for infants ≥ 35 weeks’ gestation and 21–30% for infants < 35 weeks’ gestation [49].

Among preterm infants requiring respiratory support, the definition of normoxia during the transition to extrauterine life has yet to be established, as the current O_2_ saturation targets are based on term infant data [50]. The Optimization of Saturation Targets and Resuscitation Trial (OptiSTART) is a multicenter randomized controlled trial that compares two O_2_ saturation targets during the resuscitation of preterm infants (23–30 weeks gestation). The trial hypothesizes that higher O_2_ saturation targets will lead to higher survival rates without BPD at 36 weeks PMA (NCT05849077).

## 7. O_2_ Saturation Target in Preterm Infants

O_2_ supplementation is one of the most common therapies used in neonatal intensive care units. Initially, its use was based on observations or small clinical studies [51]. The first large randomized control trial, the STOP-ROP (Supplemental Therapeutic Oxygen for Pre-threshold Retinopathy of Prematurity) trial, was published in 2000 and enrolled preterm infants with confirmed pre-threshold ROP with a mean postmenstrual age (PMA) of 35.4 weeks at enrollment. This trial compared high target O_2_ saturation (96–99%) to low O_2_ saturation (89–94%) and concluded that targeting higher O_2_ saturation was associated with worse pulmonary outcomes without the hypothesized benefits on ROP, growth, or weight gain [52].

The second large randomized controlled trial, the BOOST (Benefits of Oxygen Saturation Targeting) trial, was published in 2003 and enrolled preterm infants born at <30 weeks’ gestation at PMA of 32 weeks. This trial compared high O_2_ saturation (95–98%) with standard target O_2_ saturation (91–94%). The results were similar to those of the STOP-ROP trial, with a non-statistically significant increase in the number of deaths but prolonged O_2_ need and higher rates of home O_2_ use, therefore increasing the health system burden [53].

Following these two trials, international experts in 2003 prospectively planned a meta-analysis of five large RCTs, known as NeOProM (Neonatal Oxygenation Prospective Meta-analysis), which was published in 2018 and enrolled preterm infants born at < 28 weeks’ gestation and enrolled within 24 h of birth [54]. The RCTs included the SUPPORT trial (Surfactant Positive Airway Pressure and Pulse Oximetry) [55], published in 2010, and the BOOST II [56] and the COT [57] (Canadian Oxygen Trial) trials, published in 2013. NeOProM included a total of 4965 infants, and the results revealed that infants randomized to the lower O_2_ saturation group (85–89%) had a higher risk of death at a corrected age of 18–24 months (19.9% vs. 17.1%; *p* = 0.01) and severe necrotizing enterocolitis (9.2% vs. 6.9%; *p* = 0.003), but a lower risk of ROP treatment (10.9% vs. 14.9%; *p* < 0.003) compared to the higher O_2_ saturation group (91–95%) [58].

Based on previously published RCTs, the American Academy of Pediatrics, in their 2016 statement, suggested a targeted O_2_ saturation of 90–95% while noting that the optimal range for extremely low-birth-weight infants remains unknown [51]. The European Consensus Guidelines recommend a targeted O_2_ saturation range of 90–94% for preterm infants and suggest alarm limits of 89–95% [59].

## 8. O_2_ Saturation Target in Neonates with Bronchopulmonary Dysplasia (BPD)

Few studies have investigated the optimal O_2_ target in patients with established BPD. The infants in the STOP-ROP and BOOST trials were the closest in age to those with BPD diagnosis. The mean PMA of infants in the STOP-ROP trial was 35.4 weeks, while infants in the BOOST trial were randomized at 32 weeks. In both studies, no benefits were observed in the higher O_2_ saturation groups regarding growth and major developmental abnormalities. The STOP-ROP results showed higher rates of pneumonia, BPD exacerbation, longer O_2_ requirements, and prolonged hospitalization in the high O_2_ saturation group [52,53]. These trial results may question the benefits or even highlight the risks of higher O_2_ saturation targets in older preterm infants.

Conversely, substantial literature highlights the adverse effects of hypoxemia in infants, linking it to negative impacts on pulmonary and neurodevelopmental outcomes [60,61,62]. Additionally, among patients with BPD, an O_2_ saturation of 90% was associated with low-quality sleep, whereas O_2_ therapy was linked to prolonged REM sleep and reduced sleep interruption [63]. Growth tends to be better when targeting O_2_ saturation above 92% in patients with BPD than when maintaining O_2_ saturation between 88% and 91% [64].

The American Thoracic Society’s 2019 clinical practice guidelines on home O_2_ therapy recommend its use in patients with BPD who experience chronic hypoxemia. O_2_ therapy has been shown to improve growth, lower pulmonary arterial pressure, and enhance sleep quality and duration. According to ATS guidelines, chronic hypoxemia in patients with BPD is defined by O_2_ saturation levels maintained at or below 93%. This recommendation is graded as a strong recommendation with very low-quality evidence [7].

The Pediatric Section of the British Thoracic Society Home O_2_ Guideline Development Group endorses maintaining the same saturation goals at or above 93% [8], as does the positional statement from the Thoracic Society of Australia and New Zealand [9]. However, the European Respiratory Society’s 2019 guidelines suggest that the minimum O_2_ saturation target should be 90%. This recommendation is based on the quality of the available evidence and suggests that using a lower threshold can alleviate the financial burden on health systems and result in fewer infants being discharged on O_2_ therapy without compromising overall outcomes [10].

A recent pilot RCT involving 50 infants with established BPD, randomized to higher (≥96%) vs. lower (90–94%) O_2_ saturation target groups, found no significant difference in intermittent hypoxemia events or total time spent in hypoxemia when monitored up to 6 months of corrected age. However, post hoc analyses suggested a benefit for infants in the higher O_2_ saturation target group when the analysis was limited to data collected before 48 weeks PMA [65], which also highlights that what is needed earlier after diagnosis might not be the same thereafter. A recent editorial highlighted the potential clinical implications of these findings as a foundation for future studies; however, the long-term benefits of reducing desaturation frequency by targeting higher O_2_ saturation levels in older premature infants remain unclear [66].

Overall, there is a lack of strong evidence to support specific O_2_ saturation targets in patients diagnosed with BPD at 36 weeks gestation. Some review articles have suggested maintaining O_2_ saturation above 92% while avoiding hyperoxia above 97% [67,68]. A survey conducted among 228 NICUs in the UK, with an 80% response rate, revealed a wide range of targeted O_2_ saturations, from 80–98%. Most units targeted a range of 90–95%, while 34% targeted between 95% and 98% [69]. A randomized controlled trial in the Netherlands plans to recruit 198 preterm infants with moderate-to-severe BPD. This study aims to compare higher vs. lower O_2_ saturation limits (≥95% vs. ≥90%, respectively) with the hypothesis that infants randomized to the higher O_2_ saturation range will experience greater weight gain and lung growth [70].

## 9. O_2_ Saturation Targets in Neonates with PH

Pediatric and neonatal pulmonary hypertension are categorized into five groups according to the World Health Organization system [71]. However, efforts are ongoing to develop alternative classifications that more accurately capture the diverse clinical contributors in these populations, including prenatal, postnatal, genetic, environmental, and phenotypical factors [11,72]. This remains a significant area of research [11,73]. Identifying the etiology of neonatal pulmonary hypertension is crucial for determining optimal treatment strategies and O_2_ saturation targets in this vulnerable population.

O_2_ therapy aims to elevate the alveolar O_2_ partial pressure and alleviate hypoxic pulmonary vasoconstriction [74]. A preclinical study using lambs demonstrated that maintaining preductal O_2_ saturation targets between 95% and 99% resulted in the lowest pulmonary vascular resistance compared to targets of 90–94%, albeit with higher oxidative stress. Continuous administration of 100% O_2_ is associated with decreased pulmonary flow [75], indicating no added benefit of hyperoxia. Moreover, 100% O_2_ therapy blunts the vasodilator effect of inhaled nitric oxide and increases the risk of rebound pulmonary hypertension upon cessation of 100% O_2_ therapy [74]. Optimal pulmonary vascular resistance occurs when preductal O_2_ saturation is maintained at 90–97%, and the partial pressure of O_2_ is between 60 and 80 mmHg [5]. The interaction between O_2_ and hypoxic pulmonary vasoconstriction may be further affected by acid-base balance, as acidosis can elevate pulmonary vascular resistance and attenuate the response to O_2_ therapy.

Following birth, a successful transition to postnatal life occurs with a substantial decrease in the elevated pulmonary vascular resistance present during intrauterine life, facilitating a marked increase in pulmonary blood flow. In term infants, the pulmonary arterial pressure typically reaches levels comparable to those in adults within 2–3 months after birth [11]. Failure of this normal and expected decrease in pulmonary vascular resistance after birth leads to persistent pulmonary hypertension in newborns (PPHN). Currently, no clinical studies have examined the ideal PaO_2_ or O_2_ saturation targets for managing PPHN. Guidelines from the AHA, ATS, and European Pediatric Pulmonary Vascular Disease Network (EPPVDN) emphasize the importance of optimizing lung recruitment and O_2_ delivery while preventing intermittent hypoxia and acidosis. Regarding O_2_ therapy, the AHA and ATS suggest that high O_2_ concentrations are commonly used to counteract hypoxic pulmonary vasoconstriction, although extreme O_2_ concentrations (>60% O_2_ concentration) may be ineffective due to extrapulmonary shunting and could potentially lead to lung injury. The EPPVDN recommends supplemental O_2_ concentrations to achieve a preductal O_2_ saturation of 91–95% [12].

Preclinical studies have strongly implicated hyperoxia as a major risk factor for BPD and PH. Hyperoxia is the most commonly used insult to model BPD and PH in laboratory-based studies [76,77]. Hyperoxia causes BPD and PH through a series of mechanisms. It interrupts alveolarization and angiogenesis, reduces the vascular cross-sectional area, promotes vascular remodeling, and impairs vascular reactivity in the lungs, mainly through lung inflammation and oxidative stress. PH in preterm infants with BPD is primarily attributed to the above-mentioned pulmonary vascular abnormalities [78,79]. Similar to hyperoxia, hypoxia can also cause inflammation and oxidative stress, leading to alveolar and vascular abnormalities pathognomonic of BPD and PH. Recent clinical studies support the concept that frequent intermittent hypoxia episodes, especially when prolonged, are strongly associated with the development of BPD and PH in preterm infants [61,80]. Prolonged and severe intermittent hypoxic events are also associated with increased mortality in infants with BPD and PH. An observational cardiac catheterization study in patients with BPD and PH demonstrated that a decrease in arterial oxygen saturation abruptly increases pulmonary arterial pressure, whereas maintaining SpO_2_ between 92% and 94% significantly lowers pulmonary arterial pressure [81]. The ATS and AHA recommend using this SpO_2_ range to prevent hypoxic episodes that may contribute to the development or persistence of PH in BPD [11], while the European Society guidelines recommend targeting SpO_2_ > 93% for suspected PH and SpO_2_ > 95% for confirmed PH in patients with BPD, without specifying an upper saturation limit [12]. However, targeting SpO_2_ > 97% can be toxic to several organs, including the lungs, eyes, and brain, especially in preterm infants. These varying SpO_2_ target recommendations by these societies can stem from several factors, the major one being expert consensus playing a substantial role in the absence of high-quality data. Other influencing factors could include variations in patient populations and healthcare system priorities. Despite the existing guidance, robust research is needed to inform effective management strategies, including establishing optimal SpO_2_ targets for BPD-PH to improve patient outcomes [79]. However, the SpO_2_ targets for managing these infants should be individualized based on the clinical context, accounting for the severity of PH, degree of acidosis, Hb levels, degree and frequency of hypoxemia, and signs of tissue hypoxia [5]. Given the current evidence, the adverse effects of hypoxic episodes exceed the risk of developing or worsening ROP in infants with BPD and PH. Therefore, expert recommendations support targeting SpO2 between 92% and 95% in preterm infants with PH pending robust clinical trials and meta-analyses [5].

## 10. O_2_ Saturation and Risk for Retinopathy of Prematurity (ROP)

The correlation between O_2_ therapy and ROP progression is well documented and occurs in two phases [82,83]. Initially, early retinal hyperoxia suppresses vascular endothelial growth factor (VEGF) and inhibits angiogenesis. Subsequently, late hypoxia combined with high retinal metabolic demands prompts VEGF overexpression, triggering pathological compensatory angiogenesis [84].

O_2_ NeoProm revealed a higher incidence of treated ROP (managed with laser photocoagulation, cryotherapy, or anti-VEGF injections) among infants assigned to the higher O_2_ saturation target group than among those in the lower O_2_ saturation target group [58]. The NeoProM trials maintained a consistent O_2_ saturation target within the study population, but could adopting a changing O_2_ saturation target offer a superior approach? The rationale behind gradually increasing O_2_ saturation targets postnatally may prove beneficial in reducing the risk of ROP by avoiding early hyperoxia in phase one and late hypoxia in phase two. This alternative practical approach was suggested by a meta-analysis published in 2010, preceding the NeoProM study. While these findings offer a promising avenue, further randomized controlled trials are necessary to corroborate their efficacy and mitigate any potential adverse outcomes [85,86].

Studies have revealed a correlation between ROP development and apneic episodes necessitating bag-mask ventilation and O_2_ therapy [82]. Hyperoxia or hypoxia can generate ROS, which can lead to the destruction of the neurovascular retina [87]. The incidence of severe ROP decreases with caffeine supplementation [88]. Preterm infants born between 24 and 27 weeks gestation with a higher frequency of intermittent hypoxemia during the first 8 weeks of life are at an elevated risk of developing ROP, necessitating laser therapy [89]. Therefore, optimizing medical support and increasing the time spent within the O_2_ saturation range may result in clinical benefits. In high-income countries, ROP requiring treatment mostly affects preterm infants born before 28 weeks or weighing less than 1000 g. In low-and middle-income countries, larger preterm infants are affected by high O_2_ levels and stressors like infection and poor growth [90].

## 11. Variability in the Practice of O_2_ Saturation Target Goals

The goal of O_2_ saturation targeting in the NICU is to promote development while preventing hypoxic and hyperoxic injury associated with the under-or overuse of O_2_, respectively. These O_2_ saturation targets in the NICU have evolved based on emerging evidence.

A recent survey conducted in the United States, involving 170 NICUs across 36 states, revealed 43 different O_2_ saturation targets [91]. Similarly, a survey conducted in 27 European countries involving 193 NICUs reported 40 different saturation targets [92]. Most NICUs in both continents have revised their O_2_ saturation target policies in recent years, influenced by large randomized controlled trials and statements from the European Association of Perinatal Medicine and the American Academy of Pediatrics in 2016 [51,59]. The most utilized O_2_ saturation targets in both studies were 90–95% in over 25% of NICUs.

In addition to O_2_ saturation targets, alarm limits are used to attain these goals, introducing another layer of clinical variability and practice challenges. The most commonly used alarm limits are set two points below the lower O_2_ target range and one point above the higher O_2_ target range [91]. A study has shown that alarm limits are frequently set incorrectly, particularly the upper alarm limits, which were misconfigured over 70% of the time [93].

NICUs can standardize the alarm limits for SpO_2_ based on the limited evidence available, especially in preterm infants. These limits should be individualized based on the clinical context, typically by setting them about two percentage points below and above the target SpO_2_ range. For most preterm infants without PH or frequent or severe hypoxemia or hypoxia, a preductal SpO_2_ target between 90% and 94% is acceptable [5]; therefore, in such scenarios, the alarm limits can be set between 88% and 96%. These SpO_2_ targets are unlikely to be associated with oxidative stress and its consequences, such as lung injury/BPD and eye injury/ROP unless there is a significant systemic inflammatory response due to other reasons. However, if a preterm infant has frequent or severe hypoxemia or hypoxia or has PH due to BPD or other causes, a higher SpO_2_ target between 92% and 95% (with alarm limits set between 90% and 97%) is warranted to prevent the development and progression of PH and mortality in these patients, especially in the presence of acidosis and signs of tissue hypoxia [5]. In such scenarios, the benefits of higher SpO_2_ targets may outweigh the risks of ROP development and progression. However, if these critically ill infants are already receiving a high fraction of inspired O_2_, such as greater than 80% to 90%, a lower SpO_2_ may need to be accepted temporarily while efforts are focused on optimizing ventilation, acid-base balance, and tissue perfusion. In late-preterm infants with acute PH, animal studies support targeting a higher SpO_2_ range between 90% and 97% (with alarm limits set between 89% and 98%) to optimize pulmonary vasodilation [5]. It is also prudent to avoid SpO_2_ greater than 97% in preterm infants receiving supplemental O_2_ therapy to prevent and minimize O2 toxicity to the lungs, brain, heart, and other organs.

## 12. Intermittent Episodes of Hypoxemia and Hyperoxemia

In the NICU, O_2_ saturation is regulated by adjusting the fraction of inspired O_2_ (FiO_2_) by bedside nurses or other medical staff to maintain it within the target range. Exposure to hypoxemia is linked to adverse long-term outcomes, such as death, disability, motor impairment, and cognitive and language delays [60]. Preterm infants often encounter a high frequency of intermittent hypoxemic episodes, which are believed to be related to the degree of maturation. These episodes vary over time, with relatively few incidents during the first week of life, followed by a progressive increase from the second to the fourth week, and typically a decrease from weeks 6 to 8 [61,94].

Intermittent hypoxic events can arise from apnea or ineffective ventilation [95]. In non-ventilated infants, these episodes result from respiratory instability due to central inhibition of the drive linked to upper airway obstruction caused by insufficient activities of the upper airway dilator muscles [95]. In mechanically ventilated infants, one of the causes of these events is active exhalation, which decreases compliance and increases airway resistance, leading to atelectasis, intrapulmonary shunting, and ventilation-perfusion mismatch, ultimately resulting in hypoxemia [94].

Episodes of intermittent hypoxemia induce proinflammatory stress. Hypoxia triggers the activation of proinflammatory transcription factors, such as NF-kB and TNF-alpha [96]. Intermittent hypoxia has been linked to ROP requiring laser therapy [89]. Moreover, two post-hoc secondary analyses of the Canadian oxygen trial revealed that intermittent hypoxemia was associated with a higher risk of severe BPD [61], late death, and neurodevelopmental disabilities [60,95]. Additionally, intermittent hypoxia often leads to a return to normoxia or even hyperoxia upon the administration of supplemental O_2_, potentially resulting in intermittent hyperoxemia and subsequent oxidative stress.

Strategies such as using higher positive end-expiratory pressure, employing a volume-limited mode of ventilation, adopting prone positioning, and administering sedation have been effective in decreasing the frequency of these events [94,97]. Furthermore, the recent implementation of closed-loop automatic control of inspired O_2_ represents another potential method for further reducing the occurrence of these episodes.

## 13. O_2_ Saturation Target Utilizing Manual vs. Closed-Loop Automatic Control of Inspired O_2_

A major challenge in clinical practice is maintaining O_2_ saturation within the target range and reducing the time spent in extreme values. Several studies have shown that the targeted O_2_ saturation range was achieved only 16–64% of the time [98,99,100]. Many factors contribute to these low rates, including the nurse-to-patient ratio, education, awareness, and alarm fatigue [98,99]. Therefore, in recent years, studies have focused on using closed-loop automatic O_2_ control as a potential method to improve the time spent within the targeted O_2_ saturation ranges and reduce the extreme ranges. A meta-analysis of 13 trials involving 343 patients comparing closed-loop automatic to manual O_2_ control showed that automatic control is more efficient in maintaining O_2_ saturation within the targeted goals and preventing extreme values [101]. A similar conclusion was reached in a more recent systematic review [102]. A recent review highlighted similar results and revealed the challenges and concerns associated with this practice. To date, no study has aimed to measure clinical outcomes, which might have prevented units from acquiring expensive equipment without strong evidence. Another concern is that automatic O_2_ control may mask clinical deterioration by adjusting O_2_ supplementation and delaying more urgent medical interventions [103].

A large randomized controlled trial (RCT) aims to compare the efficacy of closed-loop automatic control of the inspiratory fraction of O_2_ to manual control among preterm infants (<28 weeks). The trial (FiO_2_-C trial) is currently enrolling patients with a planned sample size of 2340 infants (NCT03168516). This study focuses on clinical benefits and plans to evaluate clinical outcomes at 36 weeks postmenstrual age, including the rates of death, severe ROP, BPD, necrotizing enterocolitis, and neurodevelopmental delays [104,105]. However, emerging evidence suggests that integrating artificial intelligence (AI) with automation can enhance the precision of SpO_2_ targeting by individualizing O_2_ therapy based on each patient’s unique needs. AI systems can complement automated devices by predicting O_2_ needs and detecting anomalies, minimizing some of the current limitations of O_2_ therapy, such as the inability to account for perfusion and skin tone variations, patient activity, and motion artifacts [106,107]. A recent systematic review of studies incorporating AI or automated SpO_2_ systems in adults requiring long-term oxygen therapy supports the concept of synergy between these technologies, emphasizing the promising role of AI in precision medicine-based O_2_ therapy [108]. Future studies incorporating AI and automated control of inspired O_2_ are needed to enable precision-based O_2_ therapy in this vulnerable population.

## 14. Conclusions

In preterm infants, targeting higher O_2_ saturation is associated with a lower risk of mortality, but it also leads to increased ROP rates. Most guidelines adopted by various international organizations for the neonatal population are based on low levels of evidence from animal or small clinical studies and differ notably. Additionally, there is significant variability in the implementation of these guidelines within different neonatal intensive care units. The high prevalence of intermittent episodes of hypoxia and hyperoxia in the neonatal population presents significant risks, and the potential benefits of closed-loop O_2_ control in reducing these episodes are promising. In the neonatal intensive care unit, medical providers face different clinical scenarios that may require customized treatments tailored to the individual characteristics of each infant. A multidisciplinary approach is often necessary, involving multiple subspecialists, such as pulmonologists, ophthalmologists, cardiologists, and pulmonary hypertension teams. This collaboration is essential for formulating an optimal plan of care using a precision medicine approach.

## 15. Future Directions

1. Disease-or Illness-specific O_2_ saturation targets:

Further trials are needed to determine the optimal O_2_ saturation goals in preterm infants with varying degrees of PH and BPD. Furthermore, it is important to determine the optimal O_2_ saturation targets during acute illness when tissue O_2_ demand may be elevated. Additionally, new approaches should be studied, such as incorporating AI and using gradually increasing O_2_ saturation targets in preterm infants to avoid early high O_2_ exposure and late hypoxia, which might help with ROP while stabilizing and improving PH in this population.

2. O_2_ saturation targets and outcomes:

Clinical outcomes and AI studies are necessary to evaluate the potential benefits of utilizing closed-loop O_2_ control and to explore any adverse outcomes associated with the broader implementation of this approach.

3. O_2_ saturation targets and skin color:

More research is needed to determine the accuracy of SpO_2_ readings by pulse oximeters in dark-skinned infants.

4. Tissue oxygenation and O_2_ saturation targets:

Accurate noninvasive methods for monitoring tissue oxygenation are needed to complement pulse oximetry, ensuring that tissue O_2_ needs are adequately met and the primary goal of O_2_ therapy is achieved.

## Figures and Tables

**Table 1:** Factors affecting O_2_ and hemoglobin binding in the circulation: O_2_, oxygen; CO_2_, carbon dioxide; 2,3-DPG-2, 3 Diphosphoglycerate, P50-PaO_2_ at which hemoglobin is 50% saturated (26–27 mm Hg in adults and 18–19 mm Hg in neonates).

Decreased Hemoglobin Affinity for O_2_	Increased Hemoglobin Affinity for O_2_
Decrease in pH	Increase in pH
Higher temperatures	Lower temperatures
Increased CO_2_ levels	Decreased CO_2_ levels
Increased 2,3-DPG levels	Decreased 2,3-DPG levels
Adult hemoglobin vs. Fetal hemoglobin	Fetal hemoglobin vs. Adult hemoglobin
Increased O_2_ release from hemoglobin to tissues	Decreased O_2_ release from hemoglobin to tissues
Right shift of oxyhemoglobin dissociation curve	Left shift of oxyhemoglobin dissociation curve
Higher P50 (PaO_2_ required for 50% saturation is higher or lower saturation for a given PaO_2_)	Lower P50 (PaO_2_ required for 50% saturation is lower or higher saturation for a given PaO_2_)
